# Management of *Panonychus ulmi* with Various Miticides and Insecticides and Their Toxicity to Predatory Mites Conserved for Biological Mite Control in Eastern U.S. Apple Orchards

**DOI:** 10.3390/insects14030228

**Published:** 2023-02-24

**Authors:** Neelendra K. Joshi, Ngoc T. Phan, David J. Biddinger

**Affiliations:** 1 Department of Entomology and Plant Pathology, 217 Plant Sciences Bldg., University of Arkansas, Fayetteville, AR 72701, USA; 2 Fruit Research & Extension Center, Entomology, Pennsylvania State University, 290 University Dr., Biglerville, PA 17307, USA; 3 Department of Entomology, 501 Agricultural Science & Industries Building, Pennsylvania State University, University Park, PA 16802, USA

**Keywords:** ecotoxicology, European red mite, PM, pesticides, *Neoseiulus fallacis*, *Typhlodromus pyri*, *Zetzellia mali*, mite management

## Abstract

**Simple Summary:**

*Panonychus ulmi* is a pest of several agriculturally important crops, including apples. If populations are not controlled, it can cause severe foliar damage in apple that results in lower return bloom and yield loss in following year. Biological mite control of this pest can be achieved in eastern United States apple orchards if growers conserve several species of predatory mites through proper selection and timing of insecticide applications for the main pests such as codling moth which directly damage the fruit. When apple growers occasionally loose biological mite control, they need to rely on effective miticides that also conserve these predatory mite species to maintain sustainable pest mite control. Efficiency of different pesticide chemicals used for the *P. ulmi* management and their non-target effects on predatory mites conserved for biological mite control in apple orchards are discussed.

**Abstract:**

*Panonychus ulmi* (Koch) (Acari: Tetranychidae), commonly known as European red mite, is a polyphagous pest of various tree and small fruit crops, including apples. A field study was conducted to evaluate different pesticide options available for the management of *P. ulmi*, and their impact on the population of non-target predatory mite species complex consisting of *Neoseiulus fallacis*, *Typhlodromus pyri*, and *Zetzellia mali* in apple orchards. Pesticides were applied using a commercial airblast sprayer at the 3–5 mite/leaf recommended economic Integrated Pest Management (IPM) threshold or prophylactically in the spring ignoring IPM practices such as monitoring, reliance on biological control and economic thresholds. Effects on the motile and egg stages of *P. ulmi* were evaluated as were effects on the populations of predatory mites through frequent leaf counts during the season. We also recorded the subsequent overwintering eggs of *P. ulmi* from each pesticide treatment. The two prophylactic treatments containing a mixture of zeta-cypermethrin + avermectin B1 + 1% horticultural oil and abamectin + 1% horticultural oil provided effective control of *P. ulmi* population throughout the season without reducing predatory mite populations. In contrast, eight treatments applied at the recommended economic threshold of 3–5 mites/leaf were not effective in suppressing *P. ulmi* populations and most reduced predatory mites. Etoxazole had significantly higher number of overwintering *P. ulmi* eggs compared to all other treatments.

## 1. Introduction

Phytophagous mites are pests of many agricultural and ornamental crops worldwide with the most important pest species in the family Tetranychidae, which are also known as the spider mites [1,2,3,4]. One of the significant species of the phytophagous mites is the European red mite, *Panonychus ulmi* (Koch) (Acari: Tetranychidae), which is an introduced pest of nut, pome and stone fruit and some berry crops in the U.S., but a major foliar pest of apple in many apple growing regions of the world [4,5,6,7,8]. It is an established invasive species in the United States (US) where it was first reported in Oregon in 1911 [9]. *P. ulmi* has been reported from several other geographical regions and has a very wide host range [10]. This polyphagous pest infests a wide variety of economically important fruit crops, such as apple (*Malus domestica*), plum (*Prunus* spp.), peach (*Prunus persica*), pear (*Pyrus* spp.), cherry (*Prunus* spp.), as well as chestnut (*Castanea* spp.), almond (*Prunus dulcis*), and grapes (*Vitis vinifera*), etc. It has also been reported from some non-crop hosts and ornamentals such as rose [4].

*Panonychus ulmi* overwinters as eggs deposited in the rough bark, cracks and crevices of limbs and twigs of trees in apple orchards [4,11,12]. Upon favorable conditions during the following spring (which is generally between the tight cluster and bloom stages of apple), these eggs hatch into larvae that develop through protonymph and deutonymph stages to adults. Summer eggs of *P. ulmi* are generally laid on the underside of the leaves (around leaf veins), and depending on weather conditions, the species has 8–10 generations in a year in Pennsylvania apple orchards [12]. Adult and immature *P. ulmi* causes damage by primarily feeding on the host leaves and sucking their cell contents, including chlorophyll, thus interfering in the process of photosynthesis and carbohydrate production [4,13]. Continuous feeding on leaves initially causes white stippling but the damage to infested leaves quickly turns brown in what is known as ‘bronzing’ by apple growers. Depending on the population level and tree physiological stage and status, *P. ulmi* infestation in apple leaves results in economic losses by inversely interacting with crop load, fruit quality, size, and yield, as well as the bloom and development of fruit spurs (flowering and nonflowering buds) of the next year [13,14,15,16].

Population of phytophagous mites including *P. ulmi* is managed using different acaricides in conventional production. In 2013, the worldwide acaricide market was estimated to be approximately 900 million Euro, which was approximately 7% of the worldwide insecticide market value at that time [17]. Of this, it was estimated that the vast majority (74%) of acaracide chemicals were applied to fruits and vegetable crops. Because of their short life cycles and high fecundity, mites are notorious for the rapid development of pesticide resistance [18,19,20,21,22,23], which may require multiple applications of acaricides that may cost USD 125–250/ha annually if managed without biological control.

The biological mite control program using the organophosphate resistant, coccinellid predator *Stethorus punctum* (LeConte) in Pennsylvania apple orchards from the 1970s to 1995 was estimated to reduce miticide usage by 1000 metric tonnes of formulated product and saved growers approximately US 20 million over 25 years [24]. It is also thought to have delayed or prevented the development of resistance to many miticides by the European red mite, *P. ulmi* [25]. The introduction of neonicotinoid and some insect growth regulator insecticides which were toxic to *S. punctum* around 2003 in Pennsylvania saw a shift in biological mite control to the establishment and conservation of the more effective and pesticide tolerant phytoseiid mite predators (PMP) *Neoseiulus fallacis* (Garman) and *Typhlodromus pyri* (Scheuten) [24]. Over time, *T. pyri* has become the primary biological control agent of *P. ulmi* in mid-Atlantic apple orchards [26] and has been estimated to reduce acaricide use by over 80% and to have greatly reduced the development of resistance in commercial orchards [25,27]. The stigmaeid predatory mite, *Zetzellia mali* (Ewing), is also present in most commercial orchards but has low reproductive and predation rates and cannot control spider mites alone [4]. The mite control program in Pennsylvania apple orchards relies on communicating directly to growers through insecticide and acaricide efficacy guides, which is based on field trials that evaluate not only efficacy on primary pests such as codling moth, but also on safety to mite biological control and other beneficial arthropods that control secondary apple pests [12]. The well-documented case study of biological mite control in Pennsylvania apple orchards provides an example of the ways in which changes in biological mite control can happen with a shift in pesticide usage and/or the introduction of new products [25,27]. Increasingly, however, prophylactic treatments of the miticide abamectin are becoming more common in eastern apple orchards, but as in almonds, there are few studies supporting this practice [28]. Sustainable integrated pest management (IPM) practices require pest monitoring, conserving biological control, and utilization of economic spray-thresholds to prevent non-necessary sprays that may lead to increased abamectin resistance in spider mites [28].

The objectives of this research were to (a) determine whether prophylactic treatments of avermectin products early in the season are effective compared to threshold-based applications, (b) examine the efficacy of several recently introduced apple miticides on the European red mite, and (c) assess their safety to the phytoseiid predatory mite, *T. pyri*, which is currently the main component in biological mite control agent in most mid-Atlantic apple orchards and on the much less effective stigmaeid mite, *Z. mali* [12].

## 2. Materials and Methods

### 2.1. Study Orchard and Pesticide Treatments

This study was conducted in an apple orchard planted with ‘Delicious’ and ‘Yorking’ cultivars with tree spacing of ~6 × 9 m at the Penn State University Fruit Research and Extension Center, Biglerville, Pennsylvania. These cultivars were chosen as they have historically had similar susceptibility and *P. ulmi* population development. We selected ten different pesticide (acaricide/insecticide) treatments and evaluated them for control of *P. ulmi* and assessed their toxicity to the natural enemy complex of phytoseiid mite predators (PMPs—*N. fallacis* and *T. pyri*) and a stigmaeid predatory mite (*Z. mali*). The treatments were applied to single-tree plots in a randomized complete block design consisting of two replicates of ‘Delicious’ and three replicates of ‘Yorking’ cultivars which are equivalent in susceptibility to mite injury. The blocking factors were pre-counts of the levels of *P. ulmi* before treatment and cultivar for the mite threshold treatments. The trees chosen for the prophylactic treatments were completely randomized. 

### 2.2. Pesticide Treatment Application and Study Orchard Maintenance

We used a commercial airblast sprayer (Durand-Wayland airblast sprayer, Durand-Wayland, Inc., LaGrange, GA, USA) calibrated to deliver 935.37 L per hectare and driven at 3.86 km per h for a complete (i.e., both sides of tree) spray of pesticide treatments on 28 June. Eleven different insecticide/acaracide treatments and an untreated control were evaluated. A description of different pesticide treatments and their application rates is provided in Table 1. All treatments except dicloromezotiaz (#3), which is from a new mesoionic class [29], are registered for use on apple. For the purpose of brevity in the results and discussion, treatments will be referred to at first as their pesticide trade names and thereafter referred to by their treatment number or main active ingredients. In addition to these treatments, as a standard procedure to protect crop from common pests and diseases, the study orchard received a regular maintenance schedule of the following fungicides: captan (Captan^®^ 80WDG, Arysta LifeScience North America LLC, Cary, NC, USA), penthiopyrad (Fontelis^®^ 1.67L, DuPont, Wilmington, DE, USA), trifloxystrobin (Flint^®^ 50WP, Bayer CropScience, Raleigh, NC, USA), mancozeb (Manzate^®^ Pro-Stick^TM^, United Phosphorus, Inc. King of Prussia, PA, USA), myclobutanil (Rally^®^ 40WP, Dow AgroSciences, Indianapolis, IN, USA), thiophanate-methyl (Topsin^®^ M 85WDG, United Phosphorus, Inc. King of Prussia, PA, USA) and ziram (Ziram^®^ 76WP, United Phosphorus, Inc. King of Prussia, PA, USA), and the nutrient calcium chloride. In the study orchard, acetamiprid (Assail^®^ 70WP, United Phosphorus, Inc. King of Prussia, PA, USA) was applied at pink-bud stage for rosy apple aphid, *Dysaphis plantaginea* (Passerini) and after-bloom insecticides, viz. rynaxypyr (Altacor^®^ 35WDG, DuPont, Wilmington, DE, USA), flubendiamide (Belt^®^ 480SC, Bayer CropScience, Raleigh, NC, USA), and phosmet (Imidan^®^ 70WP, Gowan Company, LLC. Yuma, AZ, USA) were applied as needed (for codling moth management) at 1- to 2-week intervals beginning at petal-fall stage (early May). None of these maintenance insecticides and fungicides are known to impact pest or predatory mite populations at the time they were applied.

### 2.3. Sampling and Data Analysis

Effectiveness of the pesticide treatments on *P. ulmi* (motile mites and eggs) and their toxicity to PMP was evaluated by counting the mites at approximately weekly intervals during the season on samples of 25 leaves/tree, 125 leaves/treatment. A repeated measures analysis of variance (ANOVA) was conducted on all treatments by sample date from 21 May through the last count on 26 August. The cumulative number of mite motile days, mite egg days, PMP days, and *Z. mali* days was also determined for each summer treatment (applied on 11 July) from the first post-spray count after treatment on 15 July with multiple counts until 26 August using an area under the curve calculation as described for disease progression curves in Simko and Piepho (2012) [30]. For the two prophylactic treatments sprayed just after petal fall (22 May), cumulative number of mite days was determined from 3 June through 26 August. A subsample of PMPs was slide mounted for identification to species using a phase-contrast compound microscope using identification characters by Chant (1959) [31]. On 13 December, overwintering *P. ulmi* eggs were collected on 10 terminals per tree that were cut so the roughened growth node between new terminal growth and the old growth could be evaluated. In a 1-inch (2.5 cm) area centered around this growth node, all live *P. ulmi* eggs (red) were counted and the diameter of the twig was measured. These data were converted into *P. ulmi* eggs/cm^2^ for statistical analysis. The ANOVA was conducted on all datasets and a means separation analysis was performed using Fisher’s protected LSD test (*p* = 0.05) [32].

## 3. Results and Discussions

### 3.1. Prophylactic Avermectin Treatments

**Figure 1:**
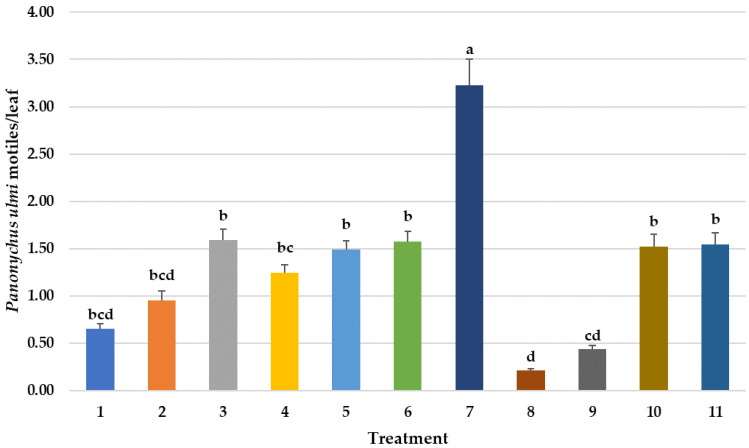
Mean number of *Panonychus ulmi* motile stages per sampled/leaf over time in different pesticide treatments. Repeated measures ANOVA–Means followed by the same letter(s) are not significantly different (Fisher’s Protected LSD, F(10,746) = 5.66, *p* < 0.001, ). Bars show the means and the error bars show standard errors. Treatment #1: cyflumetofen organo-silicon surfactant; #2: cyflumetofen surfactant; #3: dicloromezotiaz; #4: spinetoram; #5: bifenthrin; #6: fenpyroximate; #7: etoxazole non-ionic surfactant; #8: zeta-cypermethrin + avermectin B1 + mineral oil; #9: abamectin + mineral oil; #10: spirodiclofen; #11: untreated control.

**Figure 2:**
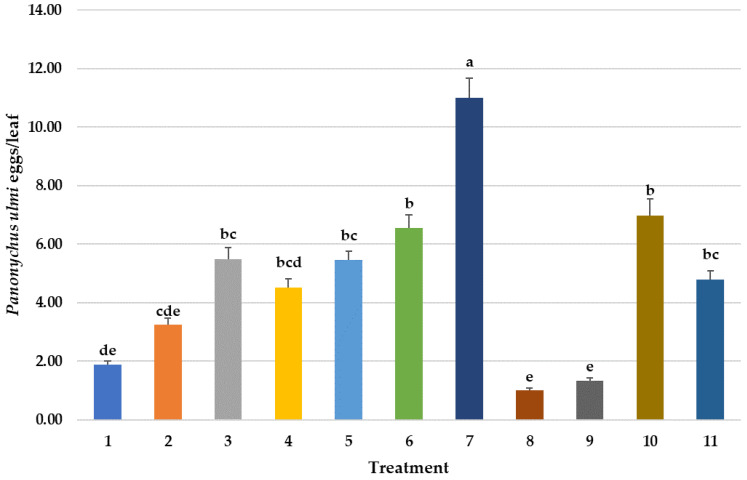
Mean number of *Panonychus ulmi* eggs per sampled/leaf over time in different pesticide treatments. Repeated measures ANOVA–Means followed by the same letter(s) are not significantly different (Fisher’s Protected LSD, F(10,746) = 9.15, *p* < 0.001). Bars show the means and the error bars show standard errors. Treatment #1: cyflumetofen organo-silicon surfactant; #2: cyflumetofen surfactant; #3: dicloromezotiaz; #4: spinetoram; #5: bifenthrin; #6: fenpyroximate; #7: etoxazole non-ionic surfactant; #8: zeta-cypermethrin + avermectin B1 + mineral oil; #9: abamectin + mineral oil; #10: spirodiclofen; #11: untreated control.

The two avermectin treatments (#8 and 9) that were applied as prophylactic treatments at the late petal fall growth stage (22 May), before pest mites had reached a spray threshold gave the best seasonal control of *P. ulmi* motile stages (Figure 1) and eggs (Figure 2) when the various counts during the season from 21 May through the last count on 26 August were evaluated using repeated measures ANOVA. Prophylactic insecticide/miticide sprays that are applied before an assessment of the pest population levels and need for control can be made are not a part of the IPM paradigm. Spraying expensive pesticides when they may not be needed will not normally save growers money or help fight the development of pesticide resistance for which *P. ulmi* is notorious for. The active ingredient, avermectin derivatives, in both treatments is not plant systemic, but it does have limited translaminar movement into the surface leaf tissues which *P. ulmi* feeds on. Unfortunately, this movement into the leaf tissues only occurs for a brief time in the spring when apple leaves are relatively new and tender, and even then, a penetrating oil or surfactant is generally needed to increase penetration into the leaf. Later in the season, when tissues harden off and develop a waxy layer to prevent desiccation, this penetration into the leaf is greatly reduced as is miticidal activity [33]. An early petal-fall, pre-spray leaf count conducted on 21 May (Figure 1) shows the typically low *P. ulmi* populations this early in the season because they are both still hatching from overwintering eggs and because very early mite instars are difficult to count. These avermectin prophylactic treatments provided significantly better control of *P. ulmi* than the remaining treatments that were applied based on the manufacturer’s labels recommending spray thresholds of 3–5 mites per leaf. These industry thresholds, however, are based on obtaining the best pest efficacy for the miticide mostly based on trials that do not have significant biological control as a factor and which do not account for variable economic mite injury thresholds that are dependent on the time of the growing season that mite injury occurs in apple.

### 3.2. Impacts on Predatory Mites

**Figure 3:**
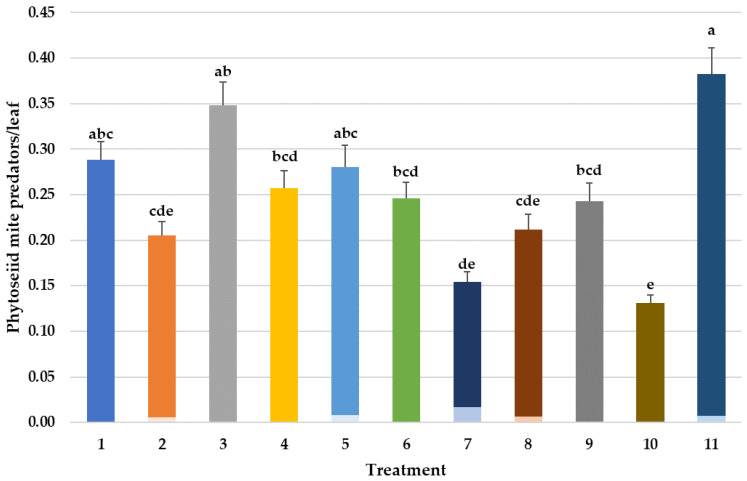
Mean abundance of phytoseiid mite predators (PMP) per sampled/leaf in various treatments over time and percent phytoseiid mite predators (PMP). Repeated measures ANOVA–Means followed by the same letter(s) are not significantly different (Fisher’s Protected LSD, F(10,746) = 3.77, *p* < 0.001). Bars show the means and the error bars show standard errors. Phytoseiid mite predators identified as *Neoseiulus fallacis* (NF) in a lighter shade at the bottom or *Typhlodromus pyri* (TP) in a darker shade on top of each column. Treatment #1: cyflumetofen organo-silicon surfactant; #2: cyflumetofen surfactant; #3: dicloromezotiaz; #4: spinetoram; #5: bifenthrin; #6: fenpyroximate; #7: etoxazole non-ionic surfactant; #8: zeta-cypermethrin + avermectin B1 + mineral oil; #9: abamectin + mineral oil; #10: spirodiclofen; #11: untreated control.

The mean abundance of phytoseiid mite predators in different treatments varied over time and it was significantly lower in the pesticide treatments 7, 8, 9, and 10 compared to control (Figure 3). Combination of different active ingredients in premix pesticides may cause more toxicity to certain groups of species, for instance, non-target predatory mites. In addition to a 28% lower rate of the active ingredient of avermectin (Table 1, 5 g ai/A vs. 6.4 g ai/A in Agri-Mek), Gladiator also has 78 g ai/A of the pyrethroid zeta-cypermethrin (Table 1). Pyrethroids can be toxic to spider mites [4] and especially to phytoseiid predatory mites [29]. Avermectin is generally more toxic to spider mites [34] than to phytoseiid predatory mites. It can be used at selective doses or at selective timings to conserve biological mite control [35]. At selective doses, avermectin-based pesticide formulations could be helpful in adjusting predator and prey ratios in apple orchards [36]. The addition of the pyrethroid zeta-cypermethrin to avermectin in the Gladiator 0.25EC (treatment #8) could have reduced predatory mites since most products in this class are generally toxic to phytoseiid predatory mites, but in this study, weekly leaf counts did not show significant reductions over time when compared to all other treatments (Figure 3). Assessing all weekly phytoseiid counts from the first pre-count on 21 May through the last count on 26 August as cumulative PMP days did, however, show significant reductions in PMPs over the control (Figure 3). Avermectin is most effective when applied early in the season before the apple leaves harden off and limit its translaminar movement into the leaf. The typical timing for applications is generally limited from the petal fall growth stage in apple to approximately 10 days later when mite populations are well below levels that can justify treatment and are difficult to count.

**Figure 4:**
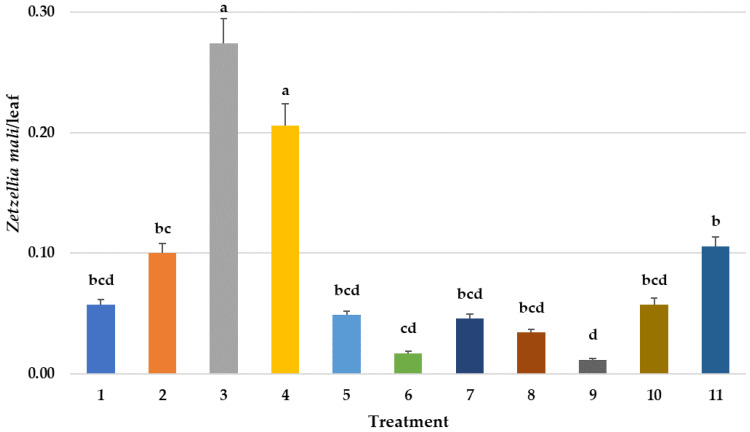
Mean abundance of stigmaeid mites *Zetzellia mali* per sampled/leaf over time in various treatments. Repeated measures ANOVA–Means followed by the same letter(s) are not significantly different (Fisher’s Protected LSD, F(10,746) = 7.06, *p* < 0.001). Bars show the means and the error bars show standard errors. Treatment #1: cyflumetofen organo-silicon surfactant; #2: cyflumetofen surfactant; #3: dicloromezotiaz; #4: spinetoram; #5: bifenthrin; #6: fenpyroximate; #7: etoxazole non-ionic surfactant; #8: zeta-cypermethrin + avermectin B1 + mineral oil; #9: abamectin + mineral oil; #10: spirodiclofen; #11: untreated control.

**Figure 5:**
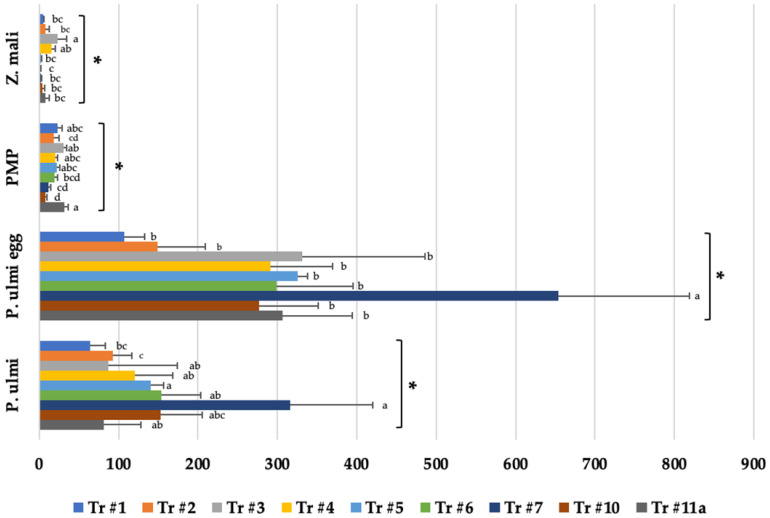
Mean number of cumulative mite days for threshold treatments. * Means followed by the same letter(s) are not significantly different (Fisher’s Protected LSD, F_*Z.mali*_(10,44) = 2.49, F_PMP_(10,44) = 2.85, F_*P.ulmi* egg_(10,44) = 3.15, F_*P.ulmi*_(10,44) = 2.32, *p* < 0.05). Bars show the means and the error bars show standard errors. Treatment #1: cyflumetofen organo-silicon surfactant; #2: cyflumetofen surfactant; #3: dicloromezotiaz; #4: spinetoram; #5: bifenthrin; #6: fenpyroximate; #7: etoxazole non-ionic surfactant; #8: zeta-cypermethrin + avermectin B1 + mineral oil; #9: abamectin + mineral oil; #10: spirodiclofen; #11: untreated control.

A more practical method for assessing the effectiveness of phytoseiid predators in orchards has been the use of predator/prey ratios that compare the weekly mite counts of both predators and *P. ulmi* prey (Table 2). Predator-to-prey ratios of 1 *T. pyri* to 10 *P. ulmi* (0.1) have been demonstrated to be an accurate predictor of biological control of this spider mite pest in apple [37]. The predator/prey ratios for both avermectin treatments were both low until mid-July, but thereafter rebounded and greatly exceeded the 0.1 ratios needed for biological control of *P. ulmi*. Predator/prey ratios for the other treatments also exceeded this ratio needed for biological control of *P. ulmi* and indicate that all treatments and the control were generally under biological control and that despite reaching an efficacy threshold established on the miticide labels, *P. ulmi* control would not have exceeded set IPM established economic thresholds as will be discussed later in the paper. The stigmaeid predatory mite *Zetzellia mali* (Ewing) is very slow, and only eats low numbers of *P. ulmi* eggs. It is generally considered unable to provide biological spider mite control by itself, although it does appear to have some tolerance to insecticides [38]. Weekly counts of this predator in all treatments were generally very low (Figure 4) and probably did not affect *P. ulmi* populations. Cumulative *Z. mali* days were not significantly different from the control except for the dichloromezotiaz treatment (#3) which was higher (Figure 5), but the more sensitive repeated measures ANOVA in Figure 4 determined reductions in the Portal and Agri-Mek treatments (#6 and 9) possibly indicating some sensitivity. In this analysis, spinetoram (#4) which has a similar mode of action to avermectin, and again the experimental dicholoromezotiaz (#3), *Z. mali* counts were significantly higher than the control (#11).

Another way of evaluating potential injury to the apple crop from *P. ulmi* population densities over time has been the use of cumulative mite days where *P. ulmi* population densities are measured over time to calculate cumulative mite days (CMD) with a recommended action threshold for spraying in Pennsylvania and New York of 500–700 CMDs [13,16]. In our study, CMD for the entire season did not reach 100 CMD even in the control (Figure 5). In the two abamectin prophylactic treatments, these IPM-based spray thresholds cannot be used, but the other treatments could have been adjusted if the predator/prey ratios had been used to determine whether biological control was working or whether interventions with miticides were needed.

### 3.3. Economic Threshold Mite Treatments (Treatments #1–7, 10)

#### 3.3.1. *Panonychus ulmi* Motiles

*Panonychus ulmi* population reached a relatively low threshold of 3–5 mites/leaf specified on most miticide labels by 8 July pre-count of the leaves and treatments were applied on 11 July. Before the applications were made, the mean number of *P. ulmi* adults was very low across all treatments in leaf samples collected in May and June. Populations of *P. ulmi* did not begin to build until 8 July in all the pre-spray counts. None of the treatments provided significant reduction in *P. ulmi* motiles (Figure 1), in contrast to the two spring treatments of avermectin (#8 and 9) that kept *P. ulmi* at very low levels. In general, pyrethroid insecticides when applied for pest management in apple orchard may disrupt conservation biological control program for *P. ulmi* due to their toxic effects on its predator *T. pyri* [39].

#### 3.3.2. Effect on *P. ulmi* Eggs

**Figure 6:**
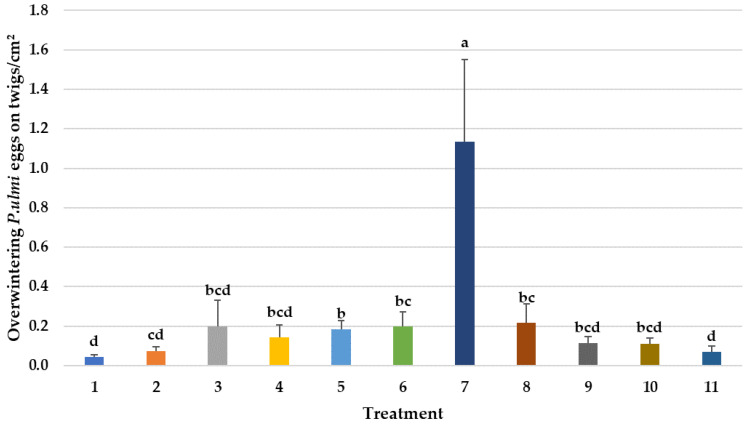
Mean number of overwintering eggs of *Panonychus ulmi* on twigs of apple trees. Means followed by the same letter(s) are not significantly different (Fisher’s Protected LSD Test, F(10,43) = 4.68, *p* < 0.001). Bars show the means and the error bars show standard errors. Treatment #1: cyflumetofen organo-silicon surfactant; #2: cyflumetofen surfactant; #3: dicloromezotiaz; #4: spinetoram; #5: bifenthrin; #6: fenpyroximate; #7: etoxazole non-ionic surfactant; #8: zeta-cypermethrin + avermectin B1 + mineral oil; #9: abamectin + mineral oil; #10: spirodiclofen; #11: untreated control.

Miticides can also affect more than one life-stages of *P. ulmi* [40]. In this study, only the two avermectin treatments and cyflumetofen (#1) significantly reduced *P. ulmi* eggs (Figure 2). Surprisingly, etoxazole (#7), which works mostly as an ovicide, had significantly higher numbers of *P. ulmi* eggs. As another measure, cumulative *P. ulmi* egg days over the entire evaluation period also determined a significantly higher number of eggs in this treatment than the control (Figure 6). Both spirodiclofen and cyflumetofen are the current grower mite control standards in mid-Atlantic apple orchards because of excellent efficacy on pest mites and minimal impact on predatory mites [12]. They have largely replaced the use of older miticides such as fenpyroximate and etoxazole which had previously developed tetranychid mite resistance [20,41]. 

### 3.4. Effects on Predatory Mites Populations

Populations of phytoseiid mite predator (PMP) species vary in their responses to different pesticides depending on the degree of resistance to different active ingredients [42]. In this study, subsamples of the PMPs from all counts in all pesticide treatments were slide mounted and identified under a phased-contrast compound microscope at 200× magnification (using keys in Chant (1959) [31]); they were almost entirely *T. pyri* and not *N. fallacis* (Figure 3). Some of the treatments (cyflumetofen + Cohere #2, etoxazole #7, both avermectin treatments #8 and 9, and spirodiclofen #10) significantly reduced populations of *T. pyri* (Figure 3), but this was not reflected in the predator/prey ratios (Table 2) starting at 4 DAT (15 July) and continuing on through July and August counts. This apparent reduction may therefore be due to changes in the *P. ulmi* prey populations as spirodiclofen has been very safe to *T. pyri* in subsequent trials by D. Biddinger. The one exception might be etoxazole (#7) which has reduced *T. pyri* in other trials by Biddinger and had the highest number of *P. ulmi* motiles and eggs of any of the treatments in this trial (Figure 1 and Figure 2). This is also reflected in the significantly higher numbers of overwintering *P. ulmi* eggs on this treatment (Figure 6) which may be due to a lack of *P. ulmi* activity if resistance is becoming an issue after almost 20 years of use and/or because of a lack of selectivity in conserving *T. pyri*. Since detected in Pennsylvania orchards almost 20 years ago [27], *T. pyri*, which, unlike other predatory mites, never leaves the apple tree for the ground cover, has been under constant selection pressure of pesticides and has currently developed a tolerance for even pyrethroids, to which it was highly susceptible early on in the crop season (D. Biddinger, unpublished data).

Some of the best-known and most cost-effective Integrated Pest Management (IPM) programs have been developed for U.S. apple orchards to conserve biological control agents that can suppress or control secondary pests such as mites, scale, leafrollers, and aphids [43]. These programs can reduce or eliminate the need for pesticide interventions and slow the development of pesticide resistance [25,44]. Primary pest such as codling moth and oriental fruit moth that feed directly on the fruit and for which there is no tolerance for damage still rely on mating disruption or insecticides [45,46,47,48] with new insecticides continually being evaluated not only for efficacy on the primary pest, but also evaluated for disruption of biological control of the secondary pests [24]. Van der Werf et al. (1994) developed predator/prey ratios to predict cumulative *P. ulmi* densities in eastern apple orchards relying on *T. pyri* demonstrated that biological mite control was almost always achieved with a ratio of 1 *T. pyri* to every 10 *P. ulmi* motile. Levels of phytoseiid predators in our trial over time (Figure 3) show that almost all predators were *T. pyri* and not the biologically different and less reliable *A. fallacis*. While some treatments reduced overall predator numbers compared to the control (Figure 3), it is the predator/prey ratios for all treatments over the sampling period (Table 2) that best demonstrate that biological control by *T. pyri* was an important factor in *P. ulmi* levels for all treatments. All treatments exceeded the 1 predator/10 *P. ulmi* (0.1) needed for biological control within 2 weeks of the 11 July application which resulted in a crash in both *P. ulmi* motile and egg populations after this time. Simplistic efficacy spray thresholds by manufacturers on their product labels also do not take into account the variable economic injury thresholds for *P. ulmi* in eastern apple orchards that change during the season due to many factors such as crop load, water stress, and age and size of the trees [13,16,49]. These have resulted in current action thresholds for Pennsylvania apple orchards of 3–5 mpl for *P. ulmi* populations before early to mid-June, 5–12 mpl for late June to early July and 8–25 mpl for mite populations after mid-July depending on the general stress trees are under due to crop load and water stress [12]. Additionally, with significant levels of *T. pyri* present (Figure 3), IPM-based spray recommendations would have raised the action threshold for spraying to approximately 8–10 mpl that late in the season. 

## 4. Conclusions

In this study, pesticide chemical treatments containing a mixture of zeta-cypermethrin and avermectin were determined to be effective in controlling *P. ulmi* population throughout the season without reducing populations of predatory mite species. Among other active ingredient chemicals, treatments containing fenpyroximate and etoxazole were not effective in reducing *P. ulmi* populations, and etoxazole had significantly higher number of overwintering *P. ulmi* eggs compared to all other pesticide treatments. Maintaining populations of predatory mite species in apple orchards is crucial in conservation biological control of *P. ulmi*. Therefore, selecting pesticides that are less toxic to predatory mites and developing strategies to mitigate non-target effects of pesticides could be helpful in maintaining predatory mite species. 

## Figures and Tables

**Table 1:** Description of pesticide treatments and application rates used for controlling *Panonychus ulmi* populations in apple orchard.

Trmt #	Treatment	Manufacturer	Active Ingredient	Amount/Acre	Grams Active Ingredient/Acre	Date of Application
1	Nealta 200SC	BASF	cyflumetofen	405 mL	81 g	11 Jul
	+Tactic	Loveland Products, Inc	organo-silicone surfactant	473 mL	--	
2	Nealta 200SC	BASF	cyflumetofen	405 mL	81 g	11 Jul
	+Cohere	Helena Agri-Enterprises, LLC	surfactant	473 mL	--	
3	DPX-RDS63 200SC	DuPont Crop Protection	dicloromezotiaz	610 mL	121.5 g	11 Jul
4	Delegate 25WG	Corteva Agriscience	spinetoram	147.4 g	36.9 g	11 Jul
5	Bifenture 2EC	United Phosphorus, Inc.	bifenthrin	379 mL	90.8 g	11 Jul
6	Portal 0.4EC	Nichino America, Inc.	fenpyroximate	946 mL	45.4 g	11 Jul
7	Zeal 72WP	Valent USA, LLC	etoxazole	85.1 g	61.2	11 Jul
	+LI-700	Loveland Products, Inc	non-ionic surfactant	473 mL (0.25% *v*/*v*)	--	
8	Gladiator 0.25EC	FMC Corporation	zeta-cypermethrin + avermectin B1	533 mL	78.0 g + 5.0 g	22 May
	+JMS Stylet Oil	JMS Flower Farms, Inc	mineral oil	3785 mL (1% *v*/*v)*	--	
9	Agri-Mek 0.15EC	Syngenta Crop Protection, LLC	abamectin	355 mL	6.4 g	22 May
	+JMS Stylet Oil	JMS Flower Farms, Inc	mineral oil	3785 mL (1% *v*/*v*)	--	
10	Envidor 2SC	Bayer CropScience	spirodiclofen	533 mL	127.8 g	11 Jul
11	Untreated Control	--	--	--	--	--

**Table 2:** Predator/Prey Ratios under various treatments.

	Predator/Prey Ratios													
Trmt #	21 May	3 Jun	11 Jun	18 Jun	24 Jun	1 Jul	8 Jul	15 Jul	22 Jul	29 Jul	5 Aug	12 Aug	19 Aug	26 Aug
1	1.0	0.1	0.1	0.0	0.2	0.0	0.1	0.1	0.3	9.0	8.0	18.0	∞	7.5
2	0.0	0.1	0.1	0.0	0.0	0.1	0.0	0.3	∞	∞	2.7	∞	∞	2.7
3	0.0	0.0	0.3	0.3	0.0	0.0	0.0	0.0	0.2	0.3	0.6	2.1	11.0	2.8
4	0.0	0.0	0.3	0.0	0.1	0.0	0.0	0.2	0.0	0.2	0.6	0.4	0.7	9.0
5	0.0	0.0	0.1	0.1	0.0	0.0	0.0	1.3	0.0	0.3	0.1	0.4	0.5	0.6
6	0.0	0.0	0.1	0.0	0.0	0.0	0.0	0.0	0.5	0.3	0.2	0.9	2.0	1.4
7	0.0	0.0	0.1	0.0	0.0	0.0	0.0	0.2	0.1	0.1	0.5	0.7	0.3	0.1
8	0.0	0.0	0.8	0.0	0.0	0.2	0.3	0.3	0.1	13.0	2.5	2.0	∞	18.0
9	0.0	0.0	1.2	0.0	0.0	0.0	0.0	3.0	1.0	9.0	2.2	2.3	5.3	∞
10	0.0	0.0	0.1	0.5	0.0	0.0	0.0	0.1	0.0	5.0	0.4	1.0	0.4	1.3
11	∞	0.0	0.4	0.0	0.0	0.1	0.0	0.1	0.3	0.9	0.6	3.5	0.5	10.0

Treatment #1: cyflumetofen organo-silicon surfactant; #2: cyflumetofen surfactant; #3: dicloromezotiaz; #4: spinetoram; #5: bifenthrin; #6: fenpyroximate; #7: etoxazole non-ionic surfactant; #8: zeta-cypermethrin + avermectin B1 + mineral oil; #9: abamectin + mineral oil; #10: spirodiclofen; #11: untreated control.

## Data Availability

Data available upon request.
